# Acute Appendicitis with Appendicolith and its Complication and Management: A Case Report

**DOI:** 10.7759/cureus.45715

**Published:** 2023-09-21

**Authors:** Maya Ann Francis, Resheek Nerella, Deepa Treesa Francis, Rohan Raj, Ahmer Zain, Sana Augustine, Niyaz A Jamil

**Affiliations:** 1 Internal Medicine, Windsor University School of Medicine, Cayon, KNA; 2 Trauma Surgery, University of Illinois College of Medicine Peoria (UICOMP), Peoria, USA; 3 Internal Medicine, Nalanda Medical College and Hospital, Patna, IND; 4 General Medicine, Kempegowda Institute of Medical Sciences, Bangalore, IND; 5 Internal Medicine, Liaquat University of Medical and Health Sciences, Jamshoro, PAK; 6 Trauma Surgery, University of Illinois/OSF St Francis Medical Center, Peoria, USA; 7 Surgery, Windsor University School of Medicine, Cayon, KNA; 8 Surgery, Combined Military Hospital, Nowshera, PAK

**Keywords:** laparoscopic technique, appendectomy, peri-appendiceal abscesses, appendicolith, acute appendicitis

## Abstract

Acute appendicitis is a common cause of acute abdominal pain requiring urgent surgery. Despite characteristic clinical signs, diagnosis can be challenging, leading to unnecessary appendectomies. This case report focuses on a 34-year-old male with escalating right lower quadrant abdominal pain. Imaging revealed acute appendicitis with a substantial appendicolith. Surgical intervention involved a open appendectomy with possible ileocecal resection due to cecal inflamation. Surgical findings indicated successful resection, and the patient recovered without complications. While urgent appendectomy is the norm, conservative approaches are gaining traction for peri-appendiceal abscesses. Interval appendectomy post-conservative treatment is a debated strategy. Management decisions are influenced by patient factors and disease severity. Future research is needed to establish standardized treatment protocols for complicated appendicitis. The case illustrates the evolving landscape of acute appendicitis management.

## Introduction

One of the common causes of acute abdomen, as well as the primary reason for immediate abdominal surgery, is acute appendicitis. Approximately 300,000 appendectomies are conducted yearly in the United States, making it the most common reason for urgent abdominal surgery [1]. Although there are well-known characteristic clinical signs and symptoms, making a quick diagnosis is not always simple because one-third of individuals are unaware of these warning signs [2,3]. It has been demonstrated that an early and accurate diagnosis can reduce the frequency of unneeded appendicectomies, which are frequently ordered due to radiological examinations [3]. The predicted lifetime probability of acute appendicitis within the United States is between 7% and 8%, with perforation rates exceeding 20% of cases [3,4]. Patients suffering from perforated appendicitis have an incidence rate of appendiceal abscess or phlegmon of approximately 3.8% [2]. Even though most patients handle surgical therapy well, there is a 2-23% chance that postoperative issues might occur [4]. Nevertheless, there is evidence that a delayed appendectomy is appropriate, and some studies have indicated that it does not correlate to a higher likelihood of complications. Without prompt treatment, some studies have shown appendicitis may advance to appendiceal perforation. Consequently, there is ongoing debate over the optimal time to have an appendectomy [5].

There is disagreement over how to treat acute appendicitis coupled with peri-appendiceal abscess. Although immediate surgery offers a conclusive course of treatment, it is technically challenging due to the adhesion of bowel loops and anatomical distortions linked with elevated intra-abdominal and wound complication rates [2,6]. According to studies, 90% of patients with abscessed perforated appendicitis can be successfully treated without surgery by administering parenteral antibiotics and maintaining close surveillance, either with or without image-guided drainage [6,7]. Furthermore, there are no established protocols for treating perforated appendicitis linked to an inflammatory mass or a peri appendiceal abscess that is not associated with widespread peritonitis. Some surgeons choose a conservative approach to treating urgent abdominal discomfort by using intravenous antibiotic therapy and performing a routine interval appendectomy (IA) after a few months [8,9]. This strategy, nevertheless, is controversial because some authors advise an urgent appendectomy regardless [8].

## Case presentation

A 34-year-old male was transferred to the emergency department from an urgent care facility due to escalating right lower quadrant (RLQ) abdominal pain that commenced the prior night. He described a one-day history of dull abdominal discomfort localized to the RLQ. Over the past day and a half, the pain had intensified. Accompanying symptoms included fever, chills, body aches, anorexia, and intermittent nausea. He had not experienced vomiting and had a regular bowel movement on the morning of the presentation. His medical history included anxiety for which he was prescribed escitalopram. He had undergone wisdom teeth extraction in the past. He reported daily nicotine and occasional alcohol consumption.

On examination, he was anxious, and his vital signs were stable with a temperature of 37.4°C, heart rate of 96 bpm, respiratory rate of 16 breaths per minute, blood pressure of 127/77 mmHg, and oxygen saturation of 98% on room air. His pain was rated 7 out of 10 in severity. Abdominal assessment revealed softness, but firm tenderness in the RLQ with mild guarding and no rebound tenderness. The rest of the physical examination did not yield any significant findings.

**Figure 1:**
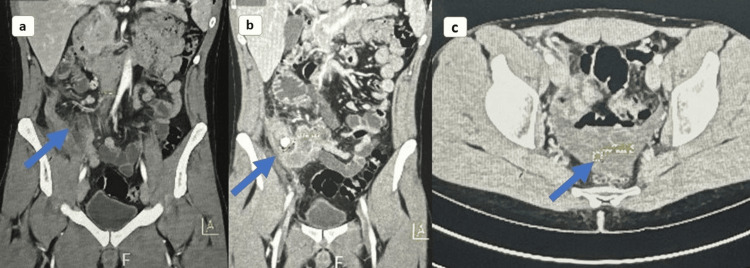
CT abdomen and pelvis, coronal (a, b) and axial section (c), showing hyper-enhancing terminal ileum of the right lower quadrant, appendicolith, and enlarged appendix.

A computed tomography (CT) of the abdomen and pelvis with IV contrast was conducted, revealing a hyper-enhancing bowel wall in the terminal ileum of the right lower quadrant and appendicolith measuring 1.2 x 1.2 cm at the origin of the appendix. The appendix itself was notably enlarged, measuring up to 1.8 cm with a thickened wall as shown in Figure 1. A mild quantity of free fluid was observed in the RLQ, without indications of loculated fluid collection.

The decision was made to admit the patient for an open appendectomy with possible ileocecal resection due to cecal inflammation. Nil per oral (NPO) orders were instituted, though he was allowed to take necessary medications. Intravenous antibiotics and fluids were initiated. Preoperative labs were conducted and confirmed negative for coronavirus disease 2019 (COVID-19). Appropriate consent for the surgical procedure was documented.

**Figure 2:**
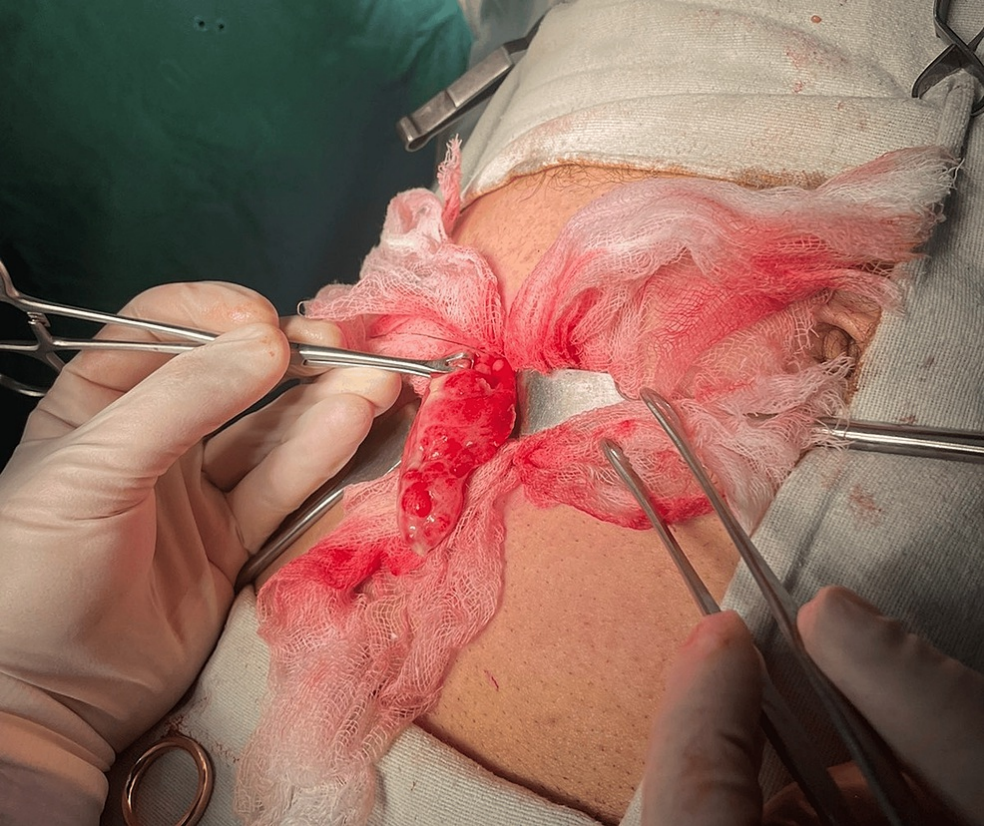
Inflamed appendix resulting from appendicolith.

During surgery under general anesthesia, he was positioned supine with proper padding. Precautions, including antibiotics and deep venous thrombosis (DVT) prophylaxis, were taken. Abdominal access was established through McBurney point using a gridiron incision. The cecal base's leathery and partial necrotic state, evident in preoperative CT scans, led to an ileocecal resection decision. Right colon mobilization preserved structures like the duodenum, while omento-colic attachments and hepatic flexure were managed skillfully. Surgical findings confirmed an inflamed appendix with severe cecal inflammation, prompting ileocecal resection (Figure 2). The terminal ileum showed no concerns. The operation proceeded smoothly without complications.

## Discussion

Luminal blockage is a cause of acute appendicitis. Foreign body impaction and lymphoid hyperplasia are the most common causes, with parasites or fecaliths being less common. A limited proportion of such cases of acute appendicitis are brought on by primary appendiceal cancers like carcinoid tumors, adenocarcinomas, Kaposi sarcomas, and lymphomas, and infrequently by metastases from breast or colon cancers. An uncommon but significant complication of appendicitis is perforation with abscess formation, which increases morbidity and mortality [9].

It is widely recognized that using CT to diagnose appendicitis has led to a significant decline in the number of unsuccessful appendectomies [1]. CT is carried out to evaluate for acute appendicitis and to distinguish it from imitating conditions like right ureteric calculus, epiploic appendicitis, torsion of a Meckel's diverticulum, mesenteric adenitis, inflammatory bowel disease, colitis, gynecological disorders, and right-sided diverticulitis [9]. Despite the use of CT scans, differentiating between non-complicated and complicated appendicitis continued to be challenging. The CT scan's significant diagnostic findings of acute appendicitis include appendiceal maximum diameter (>7 mm) and peri-appendiceal fat infiltration. Among those diagnosed with simple and complex appendicitis, the previously noted CT results, including ascites, and C-reactive protein (CRP) levels were significantly different [4].

As with many infectious diseases, appendicitis results in an increase in neutrophils and a decrease in lymphocytes; as a result, neutrophil-to-lymphocyte ratio (NLR) could be more reliable compared to white blood cell (WBC) count in determining the presence of appendicitis. As such, NLR is another easy, affordable, and straightforward diagnostic tool that can help surgeons identify acute appendicitis cases. Additionally, preoperative NLR might be used with the clinical evaluation to help differentiate between uncomplicated and severe appendicitis. Additionally, it may be utilized to evaluate patients receiving conservative treatment and those who cannot have CT scans regularly, such as pregnant women and children [3].

The presence of either an abscess, phlegm, or extraluminal gas was detected using specific CT findings to confirm the diagnosis of perforated appendicitis [1]. According to certain studies, it is challenging to diagnose appendiceal perforation or carcinoma from uncomplicated appendicitis using CT features alone [1]. Additionally, CT can diagnose postoperative intra-abdominal abscess (IAA), one of the most prevalent manifestations of appendicitis. It has also been discovered that free air was a more reliable indicator of the development of IAA than the presence of an abscess or peri-appendiceal fluid [10].

Most studies on acute, uncomplicated appendicitis indicate that urgent surgery is preferable to medical treatment. The results are worse for individuals with various comorbidities, particularly those with systemic diseases, steroid use, complex appendicitis, or those who get an urgent appendectomy. According to a recent meta-analysis on complicated appendicitis, medical care for severe appendicitis results in fewer overall sequelae, wound infections, abdominal/pelvic abscesses, and ileus/bowel obstructions [11]. Additionally, not all patients with severe appendicitis require prompt surgical intervention. Under attentive supervision, non-operative management is both practical and secure. Once the patient can be optimized, IA should be carefully considered, especially in individuals over 40 [8,11].

With clinging bowel loops and absurd anatomy, an early appendectomy is technically challenging for the treatment of a peri-appendiceal abscess or phlegmon, and the appendiceal stump tends to take longer to heal because of the presence of inflamed tissues [2]. Furthermore, percutaneous drainage is done when there is a significant or persistent collection, with antibiotic medication as the basis of treatment [9]. This initial cautious therapy is also linked to less postoperative sequelae, such as wound infections that may call for re-intervention, intraoperative visceral injury (ileal, cecal, and tubal damage), and postoperative abdominal and pelvic abscesses.

Free fecalith in abscess, which has been consistently shown to be a strong predictor leading to recurrence of acute appendicitis or abscess, has recently come to light in several investigations. Also, free fecalith should be removed when the appendiceal abscess is drained because it is the nidus of infection. Additionally, coprolith increases the likelihood of developing recurrent appendicitis, necessitating IA even after conservative therapy [8]. A few studies have also suggested that acute appendicitis is typically the cause of peri-appendiceal abscess. IA is performed in some cases to rule out the possibility of appendiceal malignancy because it might be challenging to distinguish between it and peri-appendiceal abscesses, which are also associated with appendiceal malignancy [9,12].

For cases of IA, such as the one performed on our patient, open appendectomy is recommended over laparoscopic appendectomy for better access, even though laparoscopic appendectomy is usually acknowledged to have better aesthetic results and shorter hospital stays. Additionally, when compared to open appendectomy, there is an earlier return to normal diet in laparoscopic appendectomy. However, individuals with carcinoma undergoing laparoscopic appendectomy are more likely to experience postoperative sequelae such as IAA, paralytic ileus, and intraoperative conversion to open appendectomy. Complications such as these may result from the leakage of infectious materials, the appendiceal stump not being inverted, or the resection side being exposed in the intra-abdominal space. However, additional research is needed before a standardized treatment regimen can be developed to decrease morbidity and improve outcomes for patients with perforated appendicitis and peri-appendiceal abscess.

## Conclusions

Acute appendicitis remains a significant cause of urgent abdominal surgery, posing diagnostic challenges despite well-established clinical signs. With the prevalence of appendectomies and the potential for postoperative complications, the timing and management of acute appendicitis are subjects of ongoing debate. While immediate surgery is a standard approach, conservative strategies are gaining attention for peri-appendiceal abscesses, emphasizing antibiotic therapy and IA. This case underscores the complexities in diagnosing and managing acute appendicitis, particularly cases involving abscesses or phlegmons. Imaging, mainly CT scans, is pivotal in distinguishing between simple and complicated appendicitis. Surgical techniques, such as open appendectomy, contribute to improved outcomes, but further research is needed to standardize approaches for patients with complicated presentations. Ultimately, individualized patient factors and disease severity decide treatment decisions, highlighting the evolving landscape of acute appendicitis management.
